# 

**Published:** 1988-11

**Authors:** 


Copyright © Clinical Press Ltd 1988.

### Contents

58 **250 Years of Patient Care in the Bristol Royal Infirmary**
Introduction—Prof. C. B. Perry
59 Primary Care in the Future—Prof. N. C. H. Stott.
60 Children of the Future Age—Prof. J. K. Lloyd
61 Expanding Horizons of Diagnostic Imaging—Prof. P. N. T. Wells
62 Bone Marrow Transplantation—Prof. J. R. Hobbs
63 The Vaccine Control of Virus-Induced Cancers—Prof. M. A. Epstein
64 Molecular Medicine—Prof. Sir D. J. Weatherall
65 Heart and Lung Transplantation—Mr J. Wallwork
67 Eating Tomorrow—Dr K. W. Heaton
68 **Preventive Medicine in General Practice**
Dr Jonathan Williams
71 **Trials and Tribulations with Applications for Dental Postgraduate Courses**
Dr B. G. H. Levers, Prof. C. Scully, Dr R. W. Matthews, Mr J. P. Shepherd
72 **Intestinal Fistula: A continuing Challenge: A report of the Marjorie Budd Lecture by Prof. Miles Irving**
73 **Salutary Lesson in interpretation of liver palpation at laparotomy for sigmoid carcinoma**
Mr K. R. Malpani, Mr A. Hinchcliffe, Mr S. Thomson and Dr P. Goddard
74 **From our Correspondents**—Integration of Geriatrics with General Medicine, Alzheimer's Disease, Professor John Farndon, Medicine and Retirement, Auberon Waugh on the Imprisonment of a Bristol Doctor, Inaugural Lecture in General Practice, The Bristol Medico-Historical Society, 250 years of In-patient Care at the Bristol Royal Infirmary, Overnight Sleepers
77 **Wessex Branch of the Association of Clinical Pathologists** Meeting in Bristol
79 **South West Urological Club.** Meeting at Torbay
81 Index

